# Case Report: Right Heart Failure Mistaken for Obesity—A Fault of Telemedicine

**DOI:** 10.3389/fped.2022.856911

**Published:** 2022-04-25

**Authors:** Anna Sabiniewicz, Paulina Lubocka, Robert Sabiniewicz

**Affiliations:** ^1^Medical Faculty, Medical University of Gdansk, Gdansk, Poland; ^2^Department of Pediatric Cardiology and Congenital Heart Disease, Medical University of Gdańsk, Gdansk, Poland

**Keywords:** ascending aorta, cardiac catheterization, congenital heart defects, cardiac edema, pediatric obesity, ruptured aneurysm

## Abstract

As a result of the COVID-19 pandemic, telemedicine has become an important branch of healthcare worldwide. Apart from their undeniable advantages, the virtual visits lack physical examination, which can lead to important diagnostic mistakes. We hereby present a case of a pediatric patient whose weight gain, initially attributed to a sedentary lifestyle was, in fact, due to sub-acute right heart failure in the context of a ruptured sinus of Valsalva aneurysm. The condition was not diagnosed until the patient presented at the emergency. The boy was successfully treated with two percutaneous interventions and returned to his previous stature.

## Introduction

The COVID-19 pandemic that has been overwhelming the world for the last 2 years, has led to substantial transformation of healthcare system. In order to reduce the epidemiological risk, a great part of medical appointments has been replaced by virtual visits. This solution has multiple benefits including cost-efficiency, convenience and versatility, however, lack of personal contact with the patient poses a risk of inappropriate assessment of their actual state (1). We hereby present a case of a pediatric patient, in whom a gradual weight gain caused by slowly-progressing right heart failure was wrongly taken for simple obesity.

## Case Presentation

An 11-year-old boy with autism and severe amblyopia presented to the emergency department with dyspnea, peripheral edema, and a decline in exercise tolerance. The patient was under the care of a pediatric cardiologist due to coarctation of the aorta, which was operated on in infancy, and a bicuspid aortic valve without significant dysfunction. Eighteen months prior to presentation, during routine follow-up in the department of pediatric cardiology, a computed tomography angiography (CTA) had been performed. This had revealed good long-term results of his aortic arch plasty, and dilatation of the aortic root (34 × 35 mm, *Z* score = 3.74) with a 15 × 15 mm protuberance at the right sinus of Valsalva. His scheduled follow-up had been postponed due to the COVID-19 pandemic, however his mother's opinion was that his overall condition remained stable, and they did not require any medical help. The only change reported by the family was a gradual increase in the patient's body mass, which was attributed to the COVID-19 lockdown. Indeed, the boy, who was previously overweight, had reduced his daily activity following the introduction of online education in the country. However, when his legs became edematous, his mother brought him into the emergency department. On account of the patient's cardiological comorbidities and symptoms suggesting heart failure, he was transferred to the department of pediatric cardiology.

**Figure 1:**
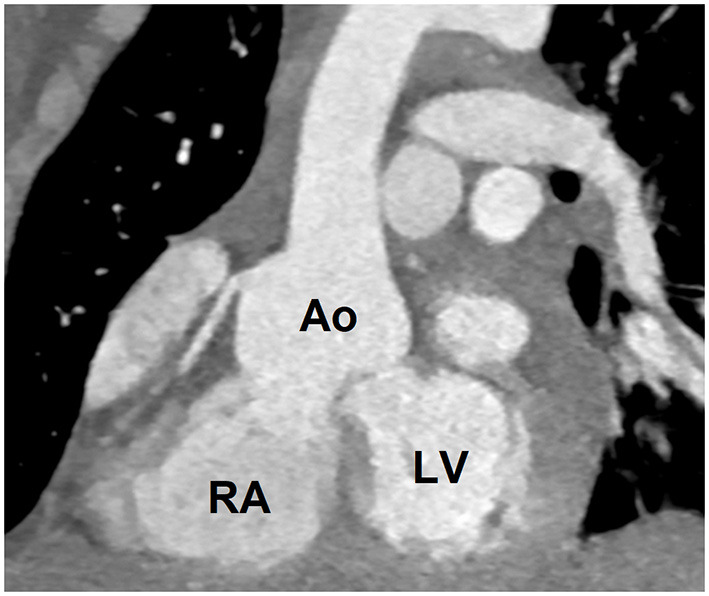
Computed tomography angiography image showing ruptured aneurysm of the right sinus of Valsalva (SVA); contrast can be seen passing from aorta to the right atrium; Ao, aorta; RA, right atrium; LV, left ventricle.

On admission, the boy's medical state was fair; he was experiencing dyspnea with little effort (NYHA class III) and was found to have sinus tachycardia up to 130 bpm, massive edema of the lower limbs and an increased waist circumference. His vital signs and basic laboratory results are summarized in Table 1. On auscultation of the chest, breath sounds were absent at the base of the lungs and there was a loud systolic-diastolic murmur (4/6 on Levine's scale). Transthoracic echocardiography (TTE) revealed right heart hyperkinesis and volume overload which was indicative of significant rupture of a sinus of Valsalva aneurysm (SVA). The diagnosis was confirmed with CTA (Figure 1).

**Table 1:** Patient's characteristics during hospital admission.

	**9.03.2020**	**17.09.2021**	**21.09.2021**	**29.10.2021**
	**(Before**	**(After**	**(After the**	**(After the**
	**rupture)**	**rupture)**	**1st**	**2nd**
			**intervention)**	**intervention)**
Height [cm]	146 (85 pc)	155 (87 pc)	155 (85 pc)	155 (85 pc)
Weight [kg]	53.6 (97 pc)	76.5 (99 pc)	70.2 (98 pc)	59.5 (95 pc)
BMI [kg/m2]	25.2 (97 pc)	31.8 (99 pc)	29.2 (98 pc)	24.8 (95 pc)
HR [bpm]	81	130	90	89
NIBP [mmHg]	144/78	155/72	144/63	130/79
NT pro-BNP [pg/ml]	<125	1,265	1,636	688

*Percentile values were calculated according to OLAF 2010 growth standards (2); pc = percentile*.

**Figure 2:**
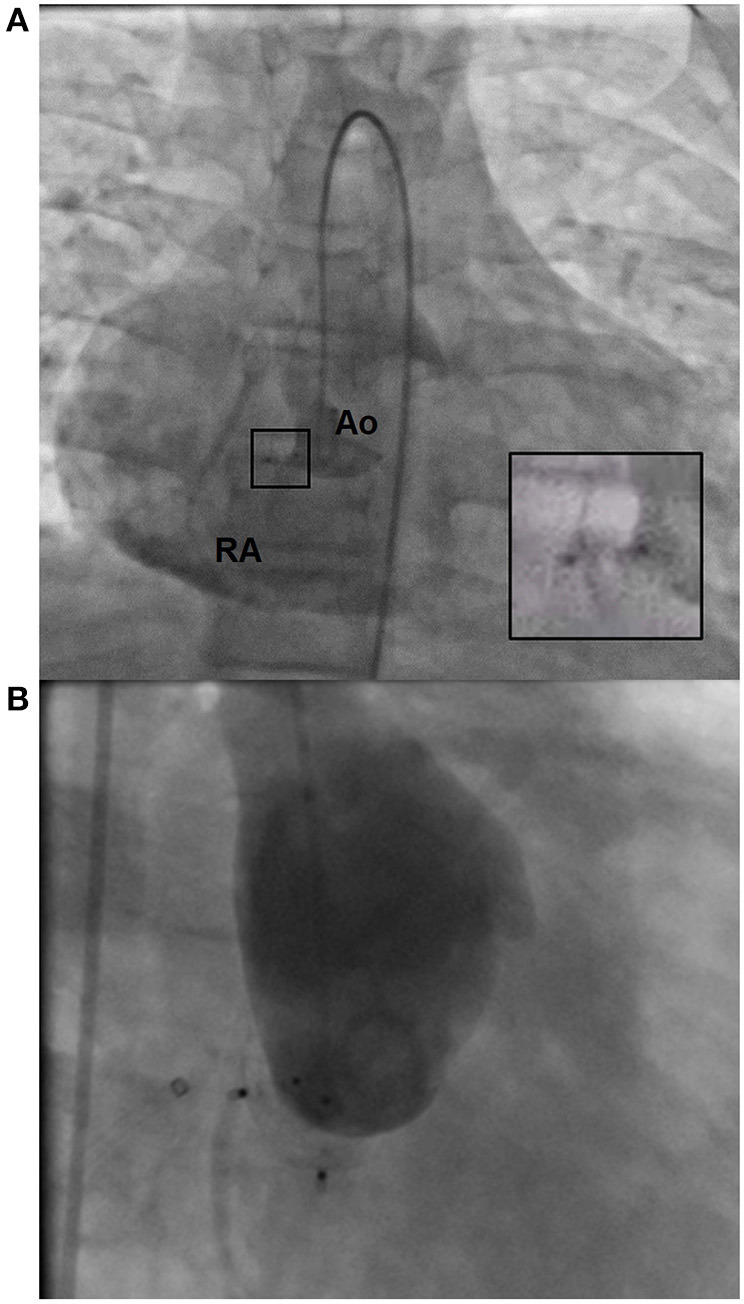
Images from cardiac catheterization. **(A)** Residual shunt following the first percutaneous intervention. The magnified image shows the ADO II device in the right SV; Ao, aorta, RA, right atrium. **(B)** Aortic root after the second intervention. The image shows two ADO II devices in place with no residual shunt to the right atrium.

The patient was admitted to hospital and treated with diuretics and antihypertensives. This resulted in a 7 kg decrease in weight, together with a reduction in edema and tachycardia. On account of infrequent character of patient's condition, an interdisciplinary case conference was held, in which percutaneous closure of the shunt was chosen as a potentially less invasive treatment strategy for a patient with multiple comorbidities. On the 4th day of hospitalization, a cardiac catheterization was performed (Figure 2A) under general anesthesia. Percutaneous access was obtained via the left femoral vein and artery, and a 6 × 4 mm Amplatzer Duct Occluder II (ADO II) device was implanted into the right sinus of Valsalva. Complete closure was not achieved at the first attempt and a residual shunt remained present above the implanted device (Figure 2A). The patient was discharged home on antihypertensives with a scheduled admission in a month. During this time, the patient's exercise tolerance improved (to NYHA class II) and his body mass remained stable. A second percutaneous intervention was performed to repair the residual shunt between the aortic root and the right atrium. The area of rupture was successfully closed with another 6 × 4 mm ADO II device (Figure 2B) with no complications. The TEE demonstrated an excellent effect of the intervention; SVA closure not only led to considerable reduction of right atrial area, but also restored respiratory variation of *vena cava* diameter and normal flow parameters through the right heart chambers. On the 2nd postoperative day the patient was discharged home. Throughout the hospital course, his body mass reduced by 17 kg as a result of diuretic therapy.

## Discussion

Systematic monitoring of body weight is an important element of routine follow-up of patients with cardiovascular conditions (3), including children with congenital heart disease (CHD). Despite frequent hospitalizations, surgical interventions, and intense rehabilitation being a risk factor for malnutrition; the prevalence of overweight and obesity in CHD patients is similar to the general population (4), however associated morbidity and mortality are much higher. Importantly, since abnormal weight gain in these patients can be associated with fluid retention, any rapid alterations in weight should raise suspicion of a worsening of their condition. SVA is an infrequent congenital heart anomaly which results from a regional defect of continuity between the tissue in the aortic wall and aortic annulus. It affects predominantly male patients (4:1) and is typically associated with ventricular septal defect (5, 6), bicuspid aortic valve (7) and aortic regurgitation (8). SVA are more prevalent in Asian populations and affect predominantly the right coronary sinus. Unruptured SVA are usually asymptomatic or mildly symptomatic, however in rare cases can they present with a heart murmur or signs of right ventricular outflow tract obstruction (9). Most cases of SVA are recognized after rupture, which typically occurs in early adulthood (13–39 years of age) (8). Considering that an SVA may be connected with the right ventricle, right atrium, pulmonary artery, pericardial sac or even the mediastinum, the clinical presentation of ruptured SVA varies depending on rapidity of the process and the affected chamber. Acute SVA rupture can lead to sudden onset right heart failure, severe aortic insufficiency or cardiac tamponade (10), however a small perforation in a dilated sinus may cause more subtle manifestations such as syncope, slow-onset shortness of breath or a gradual decline in exercise tolerance (5). In the case presented above, the timing of SVA rupture can only be presumed based on when symptoms first appeared, since the patient's home measurements of weight were not recorded. According to the parents' observations, no rapid change in his appearance was noticed. Importantly, despite the patient's previous overweight status, fluid retention was the only cause of his weight gain in the 18-month follow-up period and he otherwise maintained his percentile growth trajectory (Table 1). Aside from the subacute course of disease, there are other factors which may explain why the rupture was not discovered immediately. Firstly, the boy's autism and amblyopia impaired his ability to report his ailments at the initial stages. Secondly, he had been overweight for a long time and his routine daily activity was relatively low, so his fatigability did not raise particular concern. Finally, due to limited access to healthcare associated with the lockdown, the boy had not seen the doctor in person for a few months. A physical examination would have certainly revealed signs of heart failure and different character of heart murmur. Before the incident, our patient presented with quiet systolic murmur (2/6 Levine's scale), which changed into a loud systolic-diastolic. In this particular patient, such a phenomenon would be most probably due to newly diagnosed aortic valve regurgitation. Even though, this type of murmur can be caused by other defects, such as patent ductus arteriosus or vascular malformation, the probability of finding them in a previously operated patient is very low. Traditionally, ruptured SVA is managed with surgical intervention, including aortic valve replacement if necessary. However, an increasing number of transcatheter closures are being described (6). Despite favorable outcomes of surgical repair, transcatheter closure remains a good alternative for patients with a small ruptured area and no concomitant heart defects requiring surgery (11, 12). The prevalence of ruptured SVA is low compared to other heart defects that can be percutaneously repaired, thus, there are no commercially available devices dedicated to SVA repair. The device used in the case presented above was primarily designed to repair a patent ductus arteriosus. Other types of device have also been successfully used (6, 12, 13).

Our patient experienced SVA rupture at a relatively young age, and although he may require a cardiosurgical operation for his aortic valve and reconstructed arch in the future, management of his ruptured SVA using a transcatheter approach appeared beneficial in his case. Interestingly, the patient received two devices at two separate attempts, which is not a classic approach. Spontaneous closure of residual leaks is a commonly observed phenomenon in percutaneous interventions (14), however, since the shunt persisted and seemed to increase, we decided to perform the second procedure. To date, no concise guidelines for the management of unruptured SVA in pediatric patients have been established.

## Conclusions

Regular follow-up of patients with congenital heart disease should always include physical examination. Lack of this important element can cause a delay in making the right diagnosis and starting therapy Percutaneous closure is a promising treatment methodology for pediatric patients with ruptured SVA as it can postpone or even replace surgical intervention.

## Data Availability Statement

The original contributions presented in the study are included in the article/Supplementary Material, further inquiries can be directed to the corresponding authors.

## Ethics Statement

Written informed consent was obtained from the participant's parents/legal guardians for the publication of this case report.

## Author Contributions

PL, AS, and RS contributed to conception and design of the study. PL and AS organized the database and wrote the first draft of the manuscript. All authors contributed to manuscript revision, read, and approved the submitted version.

## Conflict of Interest

The authors declare that the research was conducted in the absence of any commercial or financial relationships that could be construed as a potential conflict of interest.

## Publisher's Note

All claims expressed in this article are solely those of the authors and do not necessarily represent those of their affiliated organizations, or those of the publisher, the editors and the reviewers. Any product that may be evaluated in this article, or claim that may be made by its manufacturer, is not guaranteed or endorsed by the publisher.
