# Supplementary figures and images for: Case Report: Right Heart Failure Mistaken for Obesity—A Fault of Telemedicine
Source: Front Pediatr. 2022 Apr 25;10:856911. doi: 10.3389/fped.2022.856911 (PMC9083262; doi:10.3389/fped.2022.856911)

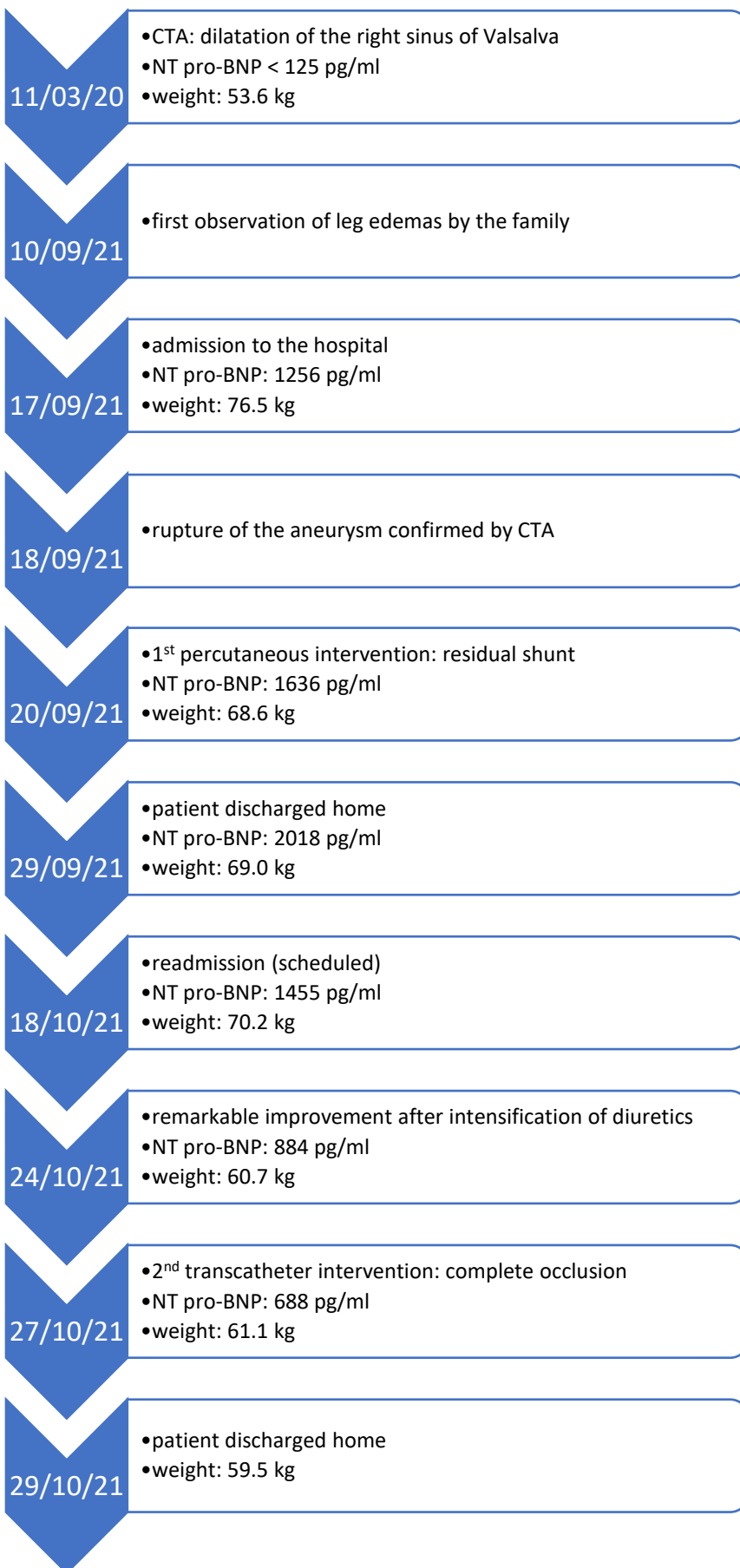

Supplement: Supplementary file 1 [file Table_1.pdf]
